# (Nano)Granules-Involving Aggregation at a Passage to the Nanoscale as Viewed in Terms of a Diffusive Heisenberg Relation

**DOI:** 10.3390/e26010076

**Published:** 2024-01-17

**Authors:** Adam Gadomski

**Affiliations:** Group of Modeling of Physicochemical Processes, Institute of Mathematics and Physics, Faculty of Chemical Technology and Engineering, Bydgoszcz University of Science and Technology, Kaliskiego 7 Street, 85-796 Bydgoszcz, Poland; agad@pbs.edu.pl

**Keywords:** matter aggregation, mesoscale, nanoscale, granule evolution, stochastic quantization, quantum-size effect, Heisenberg-type relation for the granule evolution, Fokker–Planck and diffusion-type equation, nanostructure formation

## Abstract

We are looking at an aggregation of matter into granules. Diffusion plays a pivotal role here. When going down to the nanometer scale (the so-called nanoscale quantum-size effect limit), quantum mechanics, and the Heisenberg uncertainty relation, may take over the role of classical diffusion, as viewed typically in the mesoscopic/stochastic limit. A *d*-dimensional entropy-production aggregation of the granules-involving matter in the granule-size space is considered in terms of a (sub)diffusive realization. It turns out that when taking a full *d*-dimensional pathway of the aggregation toward the nanoscale, one is capable of disclosing a Heisenberg-type (diffusional) relation, setting up an upper uncertainty bound for the (sub)diffusive, very slow granules-including environment that, within the granule-size analogy invoked, matches the quantum limit of *h*/*2πμ* (μ—average mass of a granule; *h*—the Planck’s constant) for the diffusion coefficient of the aggregation, first proposed by Fürth in 1933 and qualitatively foreseen by Schrödinger some years before, with both in the context of a diffusing particle. The classical quantum passage uncovered here, also termed insightfully as the quantum-size effect (as borrowed from the quantum dots’ parlance), works properly for the three-dimensional (*d* = 3) case, making use of a substantial physical fact that the (nano)granules interact readily via their surfaces with the also-granular surroundings in which they are immersed. This natural observation is embodied in the basic averaging construction of the diffusion coefficient of the entropy-productive (nano)aggregation of interest.

## 1. Introduction

**Figure 1:**
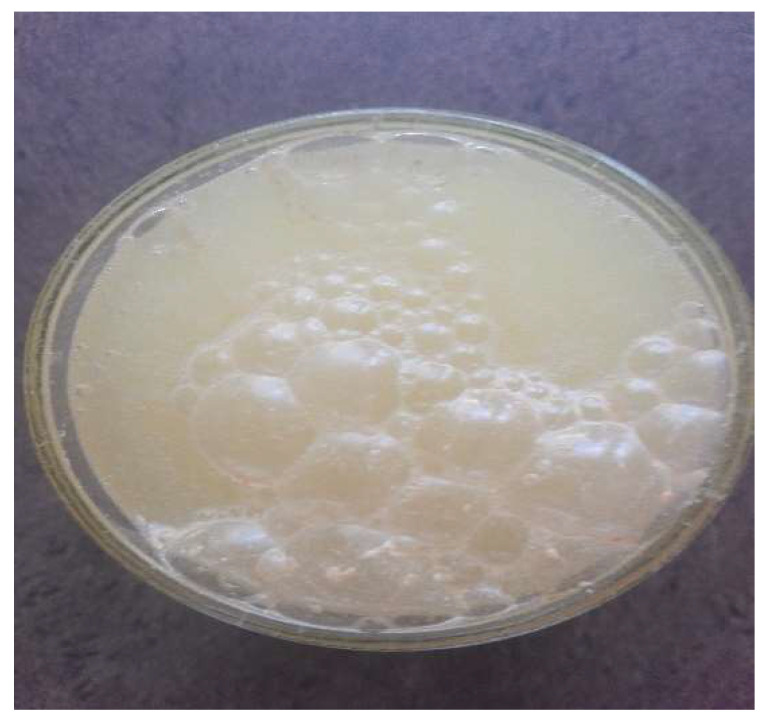
A picture of a bubbles-containing (crude) macroscopic analog system (top view), wherein the bubbles emerge in a glass full of water after some seconds when the dissolution of a soluble tablet called Arelcal 300 mg with the addition of quercine (an anti-inflammation pigment; produced by Zdrovit, Poland). The structureless granules, named bubbles here, containing a gaseous internal phase grow in a capillary mode such that the bigger (macro)granules grow in close-packing conditions at the expense of the smaller ones. (The so-called triple junctions can be identified in the picture, similarly to those observed in the grain growth [2,6,7].) The annihilation of the smaller “grains” results in having, according to the Kelvin–Laplace (capillarity) law, immensely high internal gas-phase pressures, the values of which override the surface-tension (sustainability) conditions, ultimately resulting in their blowups along with a diffusional spread of the gaseous phase to neighboring bubble(s). The system is supposed to evolve essentially in a constant-volume regime. (The vessel–wall boundary effects are postponed in further consideration).

**Figure 2:**
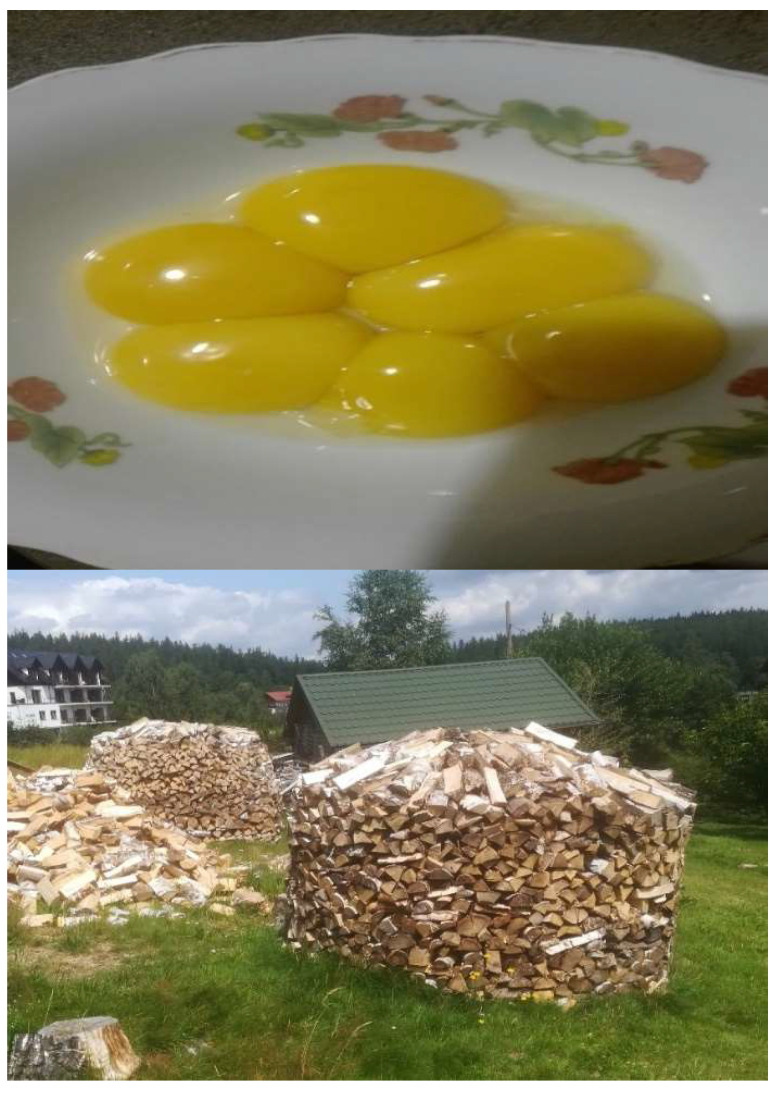
Macroscopic and taken from everyday life: illustrative (scalable) examples of sticky viz viscoelastic (with the so-called triple junctions involved) vs. non-sticky (truly granular [17]) grains/granules containing close-packed configurations from top (eggs on a plate) to bottom (pieces of wood in a gravitation-assisted pile) view, respectively.

It is widely recognized that physical systems can express their matter-aggregation-involving properties if they are formed distinctly at two different regimes. Namely, they can form either in low-temperature (quantum prone; towards (poly)crystal formation) or in high-temperature (classically meant, with amorphization effects included) limits. It is often expected that an intriguing physical scenario appears at the borderline, namely at a passage between quantum and classical expositions. This is seen when exploring a multitude of (functional) structures possessing an involvement of the so-called granules or molecular (dis)orderly tiny aggregates of versatile types. Note that throughout the paper, to avoid a certain semantic confusion, we will employ extensively the notion of granules as borrowed from Ref. [1] and applied to versatile types of aggregates (at (sub)mesoscopic) scale or agglomerates (above the mesoscopic scale; cf. Figure 1 and Figure 2). For example, polymer granules are long, repeating chains of atoms, formed via the linkage of many molecules called monomers. The monomers can be identical, or they can have one or more substituted chemical groups. Of course, these differences between monomers can affect granules’ properties such as solubility, flexibility, or mechanical stability [1,2]. To put things as simply as possible, with the notion of granules, one can identify here any, even tiny material inhomogeneity, made up of several (or, more, depending on the spatial scale [3]) atoms, molecules, oligomers, and the like.

It is commonly accepted that controlling the size and shape of nanoparticles is a challenging issue. Even though there is no external load applied on a nanoparticle or an ensemble of very small crystals, such as the quantum dots, their internal parts experience an appreciable surface stress that compensates for the corresponding capillary forces. Such a physical scenario also often shows up in colloidal self-assembly systems, especially those devoted to yielding special-purpose (soluble) nanocrystals employed in biomedical, and biomaterials science addressing applications. Functional nanocrystalline materials, made up of tiny and orderly granules, can also be mentioned in this circumstance [3,4].

There is no doubt that a challenging task emerges on a quite-general level of how to derive, from a given, viscoelastic nucleation-growth framework [1], reliable control of the size and shape of the granules, especially if the average size of them is buried in the nanoscale, i.e., in a typical range of about 1–100 nm. As one may expect, at low temperatures, and in the lower part of the distance range of a few nanometers, some quantum effects can be uncovered. But at the same size range, even in the room-temperature limit or for water at a bit lower temperature value (at about 277 K, rendering a water drop denser than in room- and/or physiological temperatures), a certain creation of bonds is feasible. However, any creation of bonds, when treated non-statistically, for example, when escaping from the reactive collisions theory, ought to be described with the help of a quantum-mechanical DFT-like framework [4,5].

In the following, we would like to introduce a mesoscopic, entropy-production model of a *d*-dimensional (soft) material formation based on the very basic rules of the evolution of grains- or granules-containing systems, wherein the granule’s volume stands for a stochastic variable (*x*), see [6] and refs. therein. The model proposed here stands for a *d*-dimensional extension of Pande’s stochastic model [6] that interconnects (through *x* $∼r^{d}$ ; *r*—grain’s radius, *d*—space dimension) the grain-boundary-involved growth rate *dr*/*dt* with the grain’s curvature *1*/*r* as manifested in a noisy thermal environment *e(t)*, according to a Langevin type of [6,7,8] *dr*/*dt* = *const.(1*/*r)* + *e(t)*.

The material system addressed is supposed to reside close to the local thermodynamic equilibrium, eventually leaving it for the neighboring one. The change in the local equilibria is eventually driven by the capillary forces (Kelvin–Laplace law: a respective pressure difference proportional to the granule’s curvature), albeit the process is not entirely deterministic because it is also driven by matter diffusion via the subsequent (soft) grain boundaries [6,7]. Qualitatively, and when referring to macroscopic scale, the process of interest, as rationalized by the scaling arguments, is supposed to be reminiscent of a bubbles-containing formation, in which curvature- and surface-tension effects tend to preserve over the corresponding spatial scales [3], see Figure 1.

In one of the previous studies, one had confined himself to a relevant circumstance, namely that it was directed towards quantum wires and very low-dimensional nano-objects, ultimately reaching the space dimension *d* = 1. As a consequence, the system has been assumed to be of the negligible role played in it typically by the surface tension [6]. In turn, to keep the needle-shaped system as a whole, one had to assume that, depending on the system of interest, the Van der Waals attraction ought to be at play while creating the structure; virtually, the electrostatic Coulomb/dipolar attraction can be of favor too. On the other hand, it qualitatively looks like we would efficiently be confined to a line (or a chain) of a granules-containing system or the like [1,8].

In the current study, we would like to take advantage of the full *d*-dimensional matter aggregation case considered elsewhere [9,10,11,12], but this time, we explore thoroughly the *x*-dependent (state-dependent) diffusion function of the aggregation in the algebraic (scalable) form of

(1) $$D=D_{o\;}x^{\alpha }$$

with

(2) $$\alpha =\frac{d-1}{d}\;,$$

where α is a characteristic surface-to-volume exponent, $D_{o\;}$ represents a diffusion constant (typically of the order of $10^{-9}\;{cm}^{2}/s$ ), and *x* $∼r^{d}$ mimics (in dimension *d*) the volume of the granule; for *x* = *v* and *d* = *3*, one would provide, for example, a sphere’s volume *v* = *(4π*/*3)* $r^{3}$ . Note that α=2/3 applies here; thus, the sphere’s surface clearly becomes *s* = *4π* $r^{2}.$ For this study, it is worth noting that the range of the linear size (radius) *r* is from sub-millimeters (at mesoscale) to nanometers. (In general, the characteristic exponent α represents a generically colloidal nature of the as-described mesoscopic aggregation.)

In the present study, we would like to focus on the following subject matter. First, we are going to explore in full the *d*-dimensional picture of the granules’ formation [9,10,12]. Second, we wish to go a step further with the stochastic quantization procedure applied to diffusion-type systems [13], proposed originally in terms of a quantum-classical crossover upon the critical dynamics, allowing us to convert the diffusion-type equation into a Schrödinger’s equation (at first, in the imaginary time domain). It is possible to obtain this if the diffusion coefficient is proportional to Planck’s constant *h* [14,15]. In the limit of *h* going to zero, i.e., while escaping from the respective quantum domain, the (soft) material evolution in *x*-space would attain a low-valued ‘subdiffusive’ (and, otherwise non-quantum [2,6]) mode, nearly causing it to trap the atoms or molecules absorbed by any of the adjacent granules [1,2,16].

However, an appreciable novelty of the current study is thought to be a Heisenberg-type diffusional relation coming out from a suitable redefinition of Equation (1), qualitatively in accord with what has been proposed in [13,14,15]. This redefinition, along with an exploration of its consequences (i.e., toward quantum-size effect [4,16]), resulting in the classical–quantum passage of the subsequent granules formation, will be presented in Section 3.

The paper, also representing a type of proof-of-concept study, is organized as follows. In Section 2, a mesoscopic model for a *d*-dimensional material formation in non-equilibrium thermodynamic and kinetic conditions is introduced [12], while in Section 3, a classical–quantum crossover of the *d*-dimensional (nano)granules-containing formation, involving the offered granule-size (*r*) dependent Heisenberg-type diffusional relation, is presented. Section 4 will contain the conclusions.

## 2. Mesoscopic Model for a Granules-Containing Formation in Non-Equilibrium Thermodynamic Conditions

Certain selected examples of some types of macro-granules-involving matter have been presented in Figure 1 and Figure 2. Of course, they are of very qualitative, and above all, of an exclusively macroscopic, and also illustrative, everyday character—an equivalent of the granule can be here as either a bubble or some liquid egg (also equipped—by analogy of the grains and bubbles—with the triple junctions; cf. Figure 2, top), or finally, as a piece of wood; thus, it is in fact a very contrasting but real granular system [17], resembling, to a certain extent, and upon sufficiently large magnification, pictures of the ensembles of nanorods under SEM or STM microscopy [18,19]. However, what they illustrate in common is that they are supposed to be close-packed structures, cf. [7], for being aware of the seminal feature of the kinetic–thermodynamic system proposed here. Notice, however, that Figure 1 and Figure 2 are somehow excused with their contents because, despite their “heavily” macroscopic character, they are designed to accommodate the constancy of the total system’s volume, cf. [1] and refs. therein. In the subsequent sections/rationale, we will refer to the close-packing conditions [7] in dimension *d* for meso- and nanoscopic matter aggregations. (Our tacit assumption for the rest of the paper’s content will be the matter-aggregation statistical scalability, cf. [6,9,10,12]).

From now on, let us introduce in short a model of the so-called normal grain growth [2,6,9,10] that is based on the physical assumption that first the system obeys the continuity equation,

(3) $$\frac{\partial f\left(x,t\right)}{\partial t}+\frac{\partial J\left(x,t\right)}{\partial x}=0.$$

To complete the problem presented in Equations (3) and (4), one has to prescribe the initial and boundary conditions, abbreviated by IBCs; the so-called delta-Dirac and absorbing IBCs can be found elsewhere [6,7,9,10]. The proposed modeling, albeit based exclusively on an analytical scheme, is consistent with other multiscale (Cauchy conditions involving) continuum conceptions of describing material formations, when passing from macro- via meso- to the nanoscale. Thus, it is consistent with the statistical scalability invoked above. In what follows, it will be demonstrated as a passage from meso-(this section) to nanoscale (next section). The idea that, at very small scales, quantum mechanics may furnish diffusion-like behavior, looks practical and worth introducing.

As anticipated in the preceding section, a decomposition of the aggregating matter fluxes *J*(*x*,*t*) into two main contributions [1,12], where

(4) $$J\left(x,t\right)=-\sigma x^{\alpha -1}f\left(x,t\right)-D_{o\;}x^{\alpha }\frac{\partial f\left(x,t\right)}{\partial x}$$

ought to be assured as a signature of colloid-type systems, namely a surface tension involving the non-gradient part (including implicitly the Kelvin–Laplace pressure term [2]) and its diffusion-type gradient-including counterpart. Bear in mind that *σ* and *D_o_* are surface tension and diffusion reference (temperature-dependent) parameters, respectively. The (skew, Weibull-type) distribution *f*(*x*,*t*) represents the probability density of finding the respective grain of size *x* at time *t*; cf. [6] and refs. therein. The characteristic exponent α reads (cf. Equation (2))

(5) $$\alpha =1-\frac{1}{d},$$

where *d*—Euclidean space dimension (*d* = 1, 2, 3 …). The skewed Weibull-type (or, in particular, Rosin-Rammler) function *f*(*x*,*t*) is, for *d* > 1, always a solution [6,9] to (4). This type of function mostly represents a subdiffusive behavior [1,2]. We realize that if *d* = 1, then *α* = 0, and in Equation (4), the diffusion-type term becomes classically defined with a constant *D_o_*. As a consequence, when using the random-walk analogy in grain sizes and coordinates spaces (*x*), one may speak of the fluctuation–dissipation relation as equivalent to the well-known Einstein–Stokes relation [8]. By it, the factor preceding the gradient of *f*(*x*,*t*) in Equation (4) becomes *x*-independent. This phenomenological (*d* = 1)-model, e.g., invented for the physical-metallurgical grains-containing system, evolving diffusively in the so-called normal grain-growth conditions (recall the mention of the IBCs above), is an entropy-production model [8]. (Notice that at *d* = 1 and for high-temperature conditions, the non-gradient term becomes small compared to its diffusion viz gradient-containing counterpart in Equation (4), as we eventually end up with the standard diffusion equation.)

However, we should be aware of the fact that non-integer values of *d* > 1 are not excluded a priori, albeit another non-integer-value-involving approach has been offered so far [1]. This approach goes beyond the classical Einstein–Stokes limit toward the subdiffusive nature of the process, requesting for a densification and/or viscosity increase over time of the grainy dynamic surroundings under consideration [2,6,8].

In general, within the idea of steering through the local flux conditions, see Equations (4) and/or (6) below, the corresponding material’s densification status looks fairly feasible. A plausible strategy can be described as follows. If the formation of the granules is driven by high temperature and it keeps a low-dimensional property (*d*~1), the system enters a pure diffusional and close-to-equilibrium regime; in turn, the entropy production tends to a minimum, and also so does the densification of the material. If the formation becomes more low-temperature oriented (causing the activation of surface tension or a capillary effect) but remains higher dimensional (*d >* 1), a countereffect of higher entropy production shows up, and the material’s densification would increase; see certain remarks below.

As a matter of fact, the *x*-independence is not the case of *d >* 1. This is because the line and/or surface tension do influence the granules’ state-dependent (and, subdiffusion-causing) evolution, driven by Equations (3) and (4) with the corresponding IBCs [2,9,10]; cf. Figure 1.

According to [7], it can be presented (cf., Equation (4)) in dimension *d*, in terms of the close-to-equilibrium entropy production, if the matter flux is written as [1,7]

(6) $$J\left(x,t\right)=-b\left(x\right)f\left(x,t\right)\frac{\partial \varphi \left(x\right)}{\partial x}-{D_{o\;}x}^{\alpha }\frac{\partial f\left(x,t\right)}{\partial x},$$

in which case the only difference, when comparing Equations (4) and (6), is that there is a free energy gradient $\frac{\partial \varphi \left(x\right)}{\partial x}$ , which implies that the coefficient (mobility) $b\left(x\right)=\frac{1}{Tf\left(x,t\right)}L\left(x\right)$ , with *L*(*x*)—an Onsager’s coefficient, see [1] for comparison. The most important physical fact [1,7] appears to be here that *b*(*x*) is inversely proportional to the local temperature *T*; thus, for the high-temperature limit, the non-diffusive term, given by the free energy gradient ( $\frac{\partial \varphi \left(x\right)}{\partial x}$ ), an irrespective of the role played by dimension *d*, vanishes. (Realize, however, that, in general, the isothermal aggregation of our concern may evolve in high/vigorous- vs. low/slow-temperature limits, and it is assumed to go at both extremes without any decisive interactions with the external bath due to its total-volume-constancy conditions; see Figure 1 and Figure 2. Of course, the entropy production of the aggregation, albeit positive, is also a decreasing function of the aggregation time [1,7].) Then, the evolution of granules is essentially based on the matter flux of Fickian form, namely

(7) $$J\left(x,t\right)=-D_{o\;}\frac{\partial f\left(x,t\right)}{\partial x},$$

with $D_{o\;}=const$ , which implies the independence (of the kinetics) of the state variable *x*, and again, the Einstein–Stokes limit is to be obeyed. The evolution of the *d* = 1-material system remains (locally) conservative, in accordance with Equation (3) for α = 0, which is equivalent to *d* = 1, cf. Equations (2) or (5). This can virtually be the case of the formations of quantum wires (with Van der Waals interactions prevailing) during a molecular beam (hetero)epitaxy process, or similarly, when, via similar nanotechnology, an emergence of nanorods proves to be efficient [19], expressing the quantum-size effect but exclusively for *d* > 1, especially if *d* = 3 appears to be the case of relevance [16,18].

## 3. Nanoscale Classical vs. Quantum Limit of the *d* = 3-Dimensional Granules-Involving Formation: Construction of the Diffusion Function

As we have already pointed out, we are interested in the matter-aggregation case for higher dimensions (*d* > 1), but with in-parallel stepping down toward quantum-fingerprint-containing [20,21] nanoscale linear dimensions of *r*-s, which is the radii of the granules/domains. Therefore, it is easy to see that the examples presented in Figure 1 and Figure 2 are of a naive and qualitative though still imaginable connotation, see the preceding text. They can, however, strengthen our intuition, at least in terms of close-packing (bio)material conditions [7].

To take an appreciable advantage of the preannounced close-packing conditions based on our matter-aggregation model, one has to resort to the evaluation of the statistical moments of the distribution *f*(*x*,*t*) [1,7] (as denoted by ${<x}^{n}(t)>:=\int x^{n}f\left(x,t\right)dx$ [22]), obeying the set of Equations (3)–(5) with the IBCs, and the *x*-dependent diffusion function *D(x)*, see Equation (1). Evaluation of the statistical moments ${<x}^{n}(t)>$ for *n* = 1, which is equivalent to obtain the total system volume *V* [22] conserved (thus, a global conservation condition applies), *V* = *const*., leads also, through ${<x}^{n=0}(t)><r\left(t\right){>}^{d}=V,\;$ to a type of subdiffusive late-time (*t*) structure-yielding behavior

(8) $$<r\left(t\right){t>}^{2}∼t^{2/(d+1)}$$

wherein the ensemble average ${<x}^{n=0}(t)>\sim t^{-d/(d+1)}$ (asymptotically) stands the average number of granules in the conservative system (1) with (3)–(5). It is acceptable that $<r\left(t\right){>}^{2}$ , cf. Equation (8), being different from the fluctuation ${<r}^{2}\left(t\right)>$ , grows a bit faster (in accordance with a temporal change in the specific volume of the aggregate) than the quantity in (8), mimicking the (averaged) area of the granule which develops in time subdiffusively for *d* > 1. For an analogy, one can consult [22] (see refs. therein too, especially [2]), in which a peculiar percolation-assisted construction of the mean-squared displacement in the space of *x*-sizes, and in analogue to Equation (8), has been offered based on the (bare) structure–property argumentation. Notice that the super-dimension *d* + 1 embodied in (8) is a quantitative signature of the close-packing measure [7,22], indicative of a minimal *d*-dimensional neighborhood of any selected grain/granule of interest [18].

However, for the quantum-size effect [16,18,23], a more clear, sophisticated procedure is desired. It can be proposed, based on the same averaging as above <…> , see ref. [24] for details, to introduce the generalized diffusion coefficient of the material formation $<\frac{d}{dt}\left(\frac{1}{\alpha }\right)x^{\alpha }\left(t\right)>$ with the characteristic colloid-type exponent α>0 . By making use of the geometrical similarity relation *x* $∼r^{d}$ (for the granule’s volume), one is able to plug it into Equations (2) or (5) and perform the differentiation over time *t*, such that

(9) $$<\frac{d}{dt}\left(\frac{1}{\alpha }\right)x^{\alpha }\left(t\right)>\sim <r(t)\frac{d}{dt}r(t)>$$

which holds true for *d* = *3* or α=2/3 . (Realize that relation (9) in its current form does not contain any information about the shape of the granules; the shape factor is assumed to be quantitatively of an order of one, being, at the same time, a dimensionless parameter, e.g., 4π/3.) Adequately defining the diffusion coefficient $D^{\left(d=3\right)}(r)$ , one is capable of providing the following Heisenberg-type diffusive relation

(10) $$D^{\left(d=3\right)}\left(r\right)≔<r(t)\frac{d}{dt}r(t)>$$

thus, in the *r*-space, meaning in the space of the (round) (nano)granules’ sizes. To our knowledge, it is a novelty and virtually a useful and practical tool for the quantum-size effect aggregations in question. It is supposed that it can be useful to test for the nanotechnologists and advanced materials experimenters able to measure [18,19] with their sophisticated devices [16], pertaining to both the size of a nanodomain and the corresponding growth attained by it. According to the commonly accepted Heisenberg-principle formulation, as translated from the coordinates’ space to the (nano)domain-size space, the more precisely one would measure the domain size, the less accurately the measurement looks of the attainable domain’s rate.

To be more specific, it is uncovered by the present feature study that if the radius (or, a linear nanogranule’s size) *r* is placed in the nanometer-scale range of the (upper) order of $10^{-7}m,$ then, by assuming $D^{\left(d=3\right)}\sim 10^{-7}\left[m\right]10^{-10}\;[m/s]$ and accepting $t\gg t_{o\;}$ as well above an initial time (fairly close to stationarity), one can obtain $D^{\left(d=3\right)}\sim 10^{-17}\left[m*\frac{m}{s}\right]=10^{-13}\left[cm*\frac{cm}{s}\right]$ . It is still within the proper range of the diffusion coefficient’s values, for example, for semiconductors, when Ga atoms perform diffusional motion along GaAs *(001)* plane [23].

A more general validation of our model with *d* > 1, and with nanoscale diffusion in granules sizes, while based on a late-time estimation of the average $<r\left(t\right)\frac{d}{dt}r\left(t\right)>,$ is the following. Namely, as evidenced via experimental as well as computer-simulation (mostly Monte Carlo [6,10]) studies for metallic, ceramic, and/or semiconductor (poly)crystals [2,9,11,18], it turns out that the granule’s radius *r(t) ~ t^s^*, with 0 < *s* < 1/2, is thus expected to go subdiffusively [1,7,22] at large enough time instants *t*. The granule’s growth rate obeys *dr(t)*/*dt~t^s^*^−1^. The product of them goes as *t*^2*s*−1^. Assuming that it is still large enough *t*-s, the average from Equation (10) is sufficiently close to the above (current) product; as in the case of a stationary nanoscale formation, one may conclude that $D^{\left(d=3\right)}$ in practice would attain a very small value such as the Planck’s constant *h* is; see the discussion below.

What we have achieved so far in striving to disclose the quantum-size effect, pertinent mostly to tiny nanoscale formations [16], is that Equation (10) can be tested readily against its quantum Heisenberg-type expression. It all can formally be performed thanks to an analogy-quantization procedure in which a profound identification between quantum fluctuations and Brownian motion is addressed, proposed by Fürth [13] in the early nineteen thirties, elaborated by Nelson in the nineteen sixties of past century [14], and later, by Ruggiero and Zanetti [15]. In this procedure, also addressed with rigor and in detail by [20,21], a derivation of Schrödinger’s equation can essentially be obtained from the Newton–Langevin type dynamics, with an aid of associated Ornstein–Uhlenbeck noise playing a role of the external medium acting on the system’s dynamics [14]. From the Fürth–Nelson quantization procedure [13,14], and also well elaborated in [15], it turns out that the diffusion coefficient included in Equation (10) gives rise to the similarity relation, namely

(11) $$D^{\left(d=3\right)}(r[nm])\;∼h$$

if one, as decisively underscored here, makes use of an effective analogy between the random walk in the *x*-space of granules’ sizes (quantum-size effect) and also that of conventional (for Brownian particles) space coordinates (*d* > 1) [22,24]; *r* belongs to the nanometer scale, *r*[*nm*], in Equation (11). Of course, Planck’s constant, *h*, conventionally viewed in terms of action (formally, Joule times second), is known as a very small quantity. It is also well accepted that converting the evolving system to the classical limit means that h→0 would apply, which is pretty consistent with the meaning of Equation (11), with very small diffusion-coefficient values [23]. The ratio of *h*/*μ*, from Equation (12) stated below, is expressed in diffusion-coefficient units, i.e., in ${cm}^{2}/s$ , see discussion below Equation (2) or that presented after Equation (10).

To exploit the size vs. space position analogy [22] in full, let us provide [20,21] the appropriate well-known expression for

(12) $$D^{\left(d=3\right)}\left(r\left[nm\right]\right)=h/2\pi \mu$$

which is first derived in [13,14] for a diffusing particle/granule of an average mass *μ*. (Realize that complete methods of solution for stochastic-classical and quantum Fokker-Planck equations have been offered by [24,25], respectively). The diffusing particle in our physical circumstance is the growing granule that is expanding in its atomic mass, and, upon the stationarity limit of the uptake with saturation effect, when a granule is ultimately being created by the addition of atoms or molecules [1,22]. Another interesting physical nanosystem would be when nanobubbles (in contrast, see Figure 1) in organic/inorganic monolayers are formed in the regime of a negligible surface tension [26], and thus, presumably along with the action of diffusive dynamics in *d* > 1 [25], Equation (7); cf. Figure 1 for a macroscopic outlook. But, looking at the impact of Equation (12) on the stationary- or late-time (saturation) limit of the essentially subdiffusive process (with a very slowly decreasing value of the diffusion coefficient), one would come to a firm quantitative conclusion that for a small-cluster (granule) mass of order of 10^−24^ kg (tens to hundreds of Ga atoms), one arrives at $D^{\left(d=3\right)}\;$ of the above indicated values in the range of the semiconductor-characteristic (GaAs) diffusion coefficients [23], cf. for some values listed after Equation (10).

## 4. Conclusions

The following conclusions can be juxtaposed as follows:

(i) The mesoscopic (principal) model offered here is essentially structureless: The only “structure” that is involved is given by the surface-to-volume (colloid-type) exponent, Equations (2) or (5), but the statistical features of the Fokker–Planck and Smoluchowski type (for its quantum analog, see ref. [27]) of the subsequent aggregational, dimension-dependent dynamics (Equations (3)–(5)) give rise to its useful, very peculiar properties;
(ii) From these dynamics, in particular, for *d* = 3, for which Equation (1) expresses its diffusion-structural behavior, it follows that the classical stochastic (diffusional) dynamics can meet their near quantum subdiffusive Heisenberg-type counterpart, contributing thoroughly to the corresponding quantum-size effect [16], thus, to obey Equations (9)–(12), what has been demonstrated in the preceding section;
(iii) It turns out that even though the mesoscopic matter aggregations do not provide any versatile structural impact, they are robust enough within the surface or interface realm of action to give rise to an efficient numerical and computer-simulational investigation toward atomic detail [6,10,18,23], based indirectly on the multiscale Cauchy-type material formation continuum-and-scalability criterion, as well as the subsequent subdiffusive behavior, represented explicitly by the Weibull statistics, for details, see [24];
(iv) As for the nanoscale aggregation, it can be affordable for the nanotechnologists and advanced materials experimenters to measure [18,19] with their sophisticated devices (STM, SEM, and AFM) [16], where both the size of a nanodomain and the corresponding growth attained by it, as immersed in the quantum Heisenberg-type diffusive-structural relation offered by this study. The (classical) Heisenberg uncertainty relation implies an indeterminacy in the spatial position. By the full analogy between the diffusions in (nano)grain- and position spaces, it would imply an indeterminacy in the grain size, provided that the grain-growth pace is well attainable, or vice versa, which is also consistent with the Fürth’s classical 1933 paper [13].

It is always to warn someone’s awareness here that, in general, an elementary entropy change, very close to local thermodynamic equilibrium (at a given temperature *T*) of the otherwise isothermal aggregation, designated by $\delta S=-\frac{1}{T}\int \mu \left(x,t\right)\delta f\left(x,t\right)dx$ as addressed by [7], depends on the chemical potential of the system *µ*(*x*, *t*), which in turn, must depend upon the elementary free system’s energy release and a chemical affinity [11]. (Note that for *T*-s high enough, one would effectively attain δS=0 ; thus, the thermodynamic equilibrium.) We think that we have to unveil δS as a useful cause of the energy win by the physical entities such as atoms or molecules as being absorbed (also, adsorbed and/or chemically bonded) by their adjacent, virtually (target-like) counterparts. In this way, for example, an effective non-equilibrium *d* > 1-structure (quantum dots) can emerge, and repetitive realization of *µ*(*x*, *t*) would rest upon the above-sketched energy-gaining and the entities’ exchange elementary process. The chemical affinity, in turn, should be realized possibly via a respective bonding [4,16]. Finally, let us also conclude that an efficient estimate for the entropy production of the granules’ formation [7], δS/δt , would require (under the above integral) a time differentiation of δf(x,t)/δt, which gives in output, according to Equations (4) or (6), one surface-tension; thus, capillarity [3] is embedded extra-term, making a clear difference between the respective (non-skewed viz symmetric) Gaussian [6,8] and (skewed, thus asymmetric) Weibull [10,24] statistics for the formations’ evolutions.

## Data Availability

Data are contained within the article.
